# Confocal Laser Endomicroscopy

**DOI:** 10.1155/2012/216209

**Published:** 2012-04-12

**Authors:** Giovanni D. De Palma, Michael B. Wallace, Marc Giovannini

**Affiliations:** ^1^Dipartimento di Chirurgia Generale, Geriatrica ed Endoscopia Diagnostica ed Operativa, Università Federico II di Napoli, Via Pansini, 5, 80131 Napoli, Italy; ^2^Department of Gastroenterology and Hepatology, Mayo Clinic, Jacksonville, FL, USA; ^3^Endoscopic Unit, Department of Medical Oncology, Paoli-Calmettes Institute, Marseille, France

Confocal laser endomicroscopy (CLE) is a newly developed endoscopic technique that enables imaging of the mucosal layer during endoscopy at a subcellular level of resolution. The method can therefore be used for the assessment of changes in vascular architecture, connective tissue, and cellular components in the mucosa, enabling endoscopists to collect real-time in vivo histological images or “virtual biopsies” of the gastrointestinal (GI) mucosa during endoscopy.

Confocal microscopy consists of focusing a laser beam (such as an argon-ion laser that generates an excitation wavelength of 488 nm, blue laser light) onto the plane of interest and filtering the returned light by means of a small pinhole which rejects out-of-focus light. The illumination and detection systems are in the same focal plane and are termed “confocal”.

After passing the pinhole, the fluorescent light is detected by a photodetection device (a photomultiplier tube or avalanche photodiode), transforming the light signal into an electrical one that is recorded by a computer. All detected signals from the illuminated spot are captured and measured. As the laser scans over the plane of interest, a whole image is obtained pixel-by-pixel and line-by-line, whereas the brightness of a resulting image pixel corresponds to the relative intensity of detected fluorescent light. The gray-scale image created is an optical section representing one focal plane within the examined specimen.

Because confocal images depend on fluorescence, a fluorescent dye (contrast agent) is required to make objects visible. The contrast agents can be applied systemically (fluorescein, tetracycline) or topically (acriflavine, cresyl violet) by using a spraying catheter. Of these, intravenous fluorescein sodium (10%) and topically applied acriflavine (0.2%) have been most commonly used in humans.

CLE can be performed currently with 2 devices: (1) integrated into an endoscope (Pentax, Tokio, Japan, herein termed eCLE) and (2) as a stand-alone probe (herein termed pCLE) capable of passage through the accessory channel of most endoscopes (Cellvizio, Mauna Kea Technologies, Paris, France).

In this special issue on CLE, we have invited a few papers that address the current potential indications for pCLE imaging in clinical gastroenterology and its potential impact in the future, particularly in the screening or surveillance of GI neoplasia.

A paper in this special issue by H. Bertani et al. reviews the role of pCLE in Barrett's esophagus for detection of esophageal dysplasia and carcinoma from a clinical practice perspective. Another paper by Mascolo et al. evaluates the accuracy of pCLE in the detection of aberrant crypt foci (ACF), comparing in double-blind manner the microendoscopic and histopathological features resulting from colonic biopsy. By pCLE, the authors identified specific crypt architecture modifications associated with changes in cellular infiltration and vessels architecture, highlighting a good correspondence between pCLE features and histology.

A paper by V. Ussui and B. Wallace reviews the role of CLE as applied to colorectal polyps detected during colonoscopy. It describes the importance of probe-based confocal endomicroscopy on colorectal polyps with a particular emphasis on distinguishing hyperplastic from neoplastic polyps.

One paper of this special issue, by F. Salvatori et al., reviews the current data on the clinical application of CLE in the study of colonic mucosa in patients with ulcerative colitis.

Finally, a paper, by R. Cannizzaro et al. describes the use of CLE to analyze the angiogenic process in colo4rectal and gastric cancer patients and the possibility of a translational approach combining the confocal imaging with the diagnosis in vivo and the specific molecular profile of the patient with the targeted antiangiogenic treatment.

*Giovanni D. De Palma* *Giovanni D. De Palma* *Michael B. Wallace* *Michael B. Wallace* *Marc Giovannini* *Marc Giovannini*

